# Assessment of a single trial impact on the amplitude of the averaged event related potentials

**DOI:** 10.3389/fncir.2023.1138774

**Published:** 2023-04-17

**Authors:** Georgy O. Fedorov, Ekaterina Levichkina, Alexandra V. Limanskaya, Marina L. Pigareva, Ivan N. Pigarev

**Affiliations:** ^1^Faculty of Engineering and Information Technology, The University of Melbourne, Parkville, VIC, Australia; ^2^Department of Optometry and Vision Sciences, The University of Melbourne, Parkville, VIC, Australia; ^3^Institute for Information Transmission Problems (Kharkevich Institute), Moscow, Russia; ^4^Institute of Higher Nervous Activity and Neurophysiology, Moscow, Russia

**Keywords:** event related potentials (ERP), evoked potentials, trial-by-trial analysis, EEG, LFP, response variability

## Abstract

Widely used in neuroscience the averaging of event related potentials is based on the assumption that small responses to the investigated events are present in every trial but can be hidden under the random noise. This situation often takes place, especially in experiments performed at hierarchically lower levels of sensory systems. However, in the studies of higher order complex neuronal networks evoked responses might appear only under particular conditions and be absent otherwise. We encountered this problem studying a propagation of interoceptive information to the cortical areas in the sleep-wake cycle. Cortical responses to various visceral events were present during some periods of sleep, then disappeared for a while and restored again after a period of absence. Further investigation of the viscero-cortical communication required a method that would allow labeling the trials contributing to the averaged event related responses–“efficient trials,” and separating them from the trials without any response. Here we describe a heuristic approach to solving this problem in the context of viscero-cortical interactions occurring during sleep. However, we think that the proposed technique can be applicable to any situation where neuronal processing of the same events is expected to be variable due to internal or external factors modulating neuronal activity. The method was first implemented as a script for Spike 2 program version 6.16 (CED). However, at present a functionally equivalent version of this algorithm is also available as Matlab code at https://github.com/george-fedorov/erp-correlations.

## Introduction

It is hard to find a method as often used in neuroscience as the averaging of event related potentials (ERP). This method was introduced into electrophysiology by Barlow (1959), and is based on the assumption that each single trial waveform is the combination of a relatively constant neural response to a stimulus and a variable random noise superimposed on it. As a result of averaging procedure signal-to-noise ratio is growing and a signal shape becomes sufficiently prominent for the analysis (Dawson, 1954; Picton et al., 1995). However, in reality the components resulting from neuronal activity might vary in latency or shape due to stochastic nature of neuronal signaling and other sources of variability, e.g., variation of blood supply to a tissue under the recording electrode. In some studies, these minor variations can be ignored. However, in some cases they may influence the results to the point of changing its meaning [discussed, e.g., in Truccolo et al. (2002)], and might reflect specific network state phenomena (Kelly et al., 2010). Huffmeijer et al. (2014) assessed the reliability of different ERP component and revealed that components might have substantially different levels of repeatability, ranging from excellent to poor. The problem of inter-trial (trial-by-trial) variability has been recently reviewed by Trenado et al. (2019). The authors have pointed out that response variability changes in various psychiatric disorders as well as with normal aging and during behavioral adjustments to the task requirements. Thus, the variability might potentially serve as a functional marker of cognitive processing.

We encountered this problem in our own sleep studies focused on testing predictions of the visceral theory of sleep. This theory proposes that neurons in the sensory cortical areas, which in wakefulness respond to exteroceptive and proprioceptive stimulation, during sleep “switch” to processing of interoceptive stimuli (e.g., coming from gastrointestinal system, heart, respiration, etc.) (Pigarev, 2014), as propagation of the visceral signals to the cortex, e.g., judged by the amplitude of evoked responses to visceral stimuli, is more effective in sleep (e.g., Pigarev, 1994; Levichkina et al., 2021; Rembado et al., 2021). We have also encountered similar changes associated with state of vigilance in the dynamic of somatic and visceral signal transmissions in the ascending somatovisceral fibers in the spinal cord (Levichkina et al., 2022).

We have studied cortical evoked responses recorded from various cortical sensory areas to electrical (Pigarev, 1994; Levichkina et al., 2021) or magnetic (Pigarev et al., 2008) stimulation applied to different visceral organs in cats, monkeys and rabbits. However, we also analyzed the relationships between naturally occurring periodic events in the activity of the studied visceral organs and cortical activity, e.g., used as triggers for averaging neuronal activity maxima of the R wave in ECG, or the elements of periodic myoelectric activity of duodenum or stomach associated with peristaltic waves, and found that slow wave sleep drastically increased the probability of such gut-brain relationships (Pigarev et al., 2013).

Conducting these studies we noticed that during sleep the propagation of interoceptive information to the cortical areas was not constant. Quite often prominent cortical responses were present during some periods of sleep, then disappeared for a while and reappeared again after a period of absence. This temporal pattern of viscero-cortical interaction occurring during sleep attracted our attention. However, evoked cortical responses to visceral stimuli, as well as responses to exteroceptive sensory stimulation in wakefulness, were relatively small in a single trial and could be clearly visualized only after averaging across multiple trials. Therefore, further investigation of the viscero-cortical communication in the sleep-wake cycle required a method that would allow labeling the trials contributing to the averaged evoked responses. We further refer to them as the “efficient trials.” The problem is not limited to evaluation of naturally occurring responses, but also extends to situations where some external stimulation is applied or perception can be interrupted by variation of the internal state, which is not uncommon in neuroscience. One striking example of such internal variability of information transfer is a phenomenon of local or partial sleep, when certain brain areas transition into sleep while the animal’s behavior demonstrates overall awareness and the ability to respond to presented stimuli (Pigarev et al., 1997; Rector et al., 2005; Vyazovskiy et al., 2011).

In this article we describe our heuristic approach to solving this problem in the context of viscero-cortical interactions occurring during sleep. However, we think that the proposed method can be applicable not only to sleep research, but to any situation when neuronal processing is expected to be variable, e.g., to visual processing under natural viewing conditions or neuronal activity influenced by variation of attention paid to stimuli by a subject. The method was first implemented as a script for Spike 2 program version 6.16 (Cambridge Electronic Design). However, at present a functionally equivalent version of this algorithm is also implemented as Matlab code available at https://github.com/george-fedorov/erp-correlations.

In order to demonstrate this method of data analysis we utilized recordings obtained from New Zealand rabbits in a methodologically similar way to our previous work described in detail in Levichkina et al. (2021). Results of that study, focused on gut-brain interactions, were previously reported (Pigarev et al., 2004). No new animal or human data were collected for this article. The method included the following three steps.

At the first step of data analysis we used a conventional procedure of averaging of the cortical evoked responses to repetitive stimuli. We marked all Positive (upward) and Negative (downward) waves of the obtained evoked responses as P and N with numbers reflecting latencies of their maxima or minima correspondingly. For all obtained components of ERPs we tested statistical significance of these deflections (the procedure is explained in detail later).

As each of the significant waves could reflect activity of different functional groups of neurons responsible for different aspects of information processing, each of them could potentially be evoked only by some of the applied stimuli and not by all of the stimuli equally. Thus, these ERP components could be unrelated in a sense that each one can occur independently from the others.

The second step of the analysis included selection of the component of interest for which the “efficient” trials needed to be identified. The shape of this component, located within particular time window after stimulation, was used as a template for identification of the “efficient” trials. The idea of using templates to find segments of a signal similar to particular shape was expressed early on by Woody (1967) in the concept of *adaptive filter*. The goal of a template-based approach is to evaluate the degree of likeness of an individual trial and the template.

We estimated a similarity of every single trial waveform to the template of the ERP component in question. The estimation could be performed using different similarity thresholds to extract individual trials having different levels of contribution to the activity shaping this particular component of the response.

At the last step we averaged waveforms of the selected efficient trials, and compared their shapes to the ones obtained after averaging of all residual “inefficient” trials. In order to find an optimal threshold level for trial separation we repeated this procedure, identifying efficient and residual trials for different categorization thresholds. Similar procedure could be applied to each component of the evoked potential. This procedure is described in detail below.

## Materials and methods

### Cortical evoked responses and estimation of their significance

**FIGURE 1:**
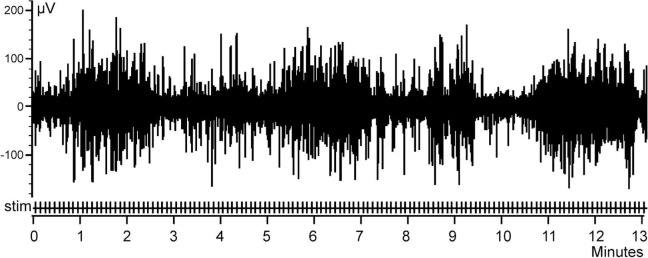
An example of a rabbit electroencephalogram (EEG) activity. Stimulations of the abdominal viscera are denoted as Stim.

**FIGURE 2:**
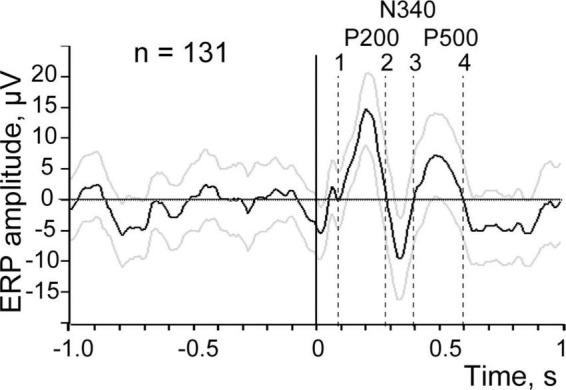
Evoked response to visceral stimulation averaged across the entire recording session, the averaging is triggered by all stimulation (*n* = 131). Black line demonstrates the mean amplitude, gray lines signify ± 2 SEM. Positive ERP components are marked by P and the negative one by N. Vertical dashed lines show time intervals used for further analysis.

As mentioned above, for test purposes we utilized electroencephalogram (EEG) recordings collected in our previous study using epidural bipolar electrodes located over the visual cortex of a rabbit during natural slow wave sleep. In that experiment, intraperitoneal electrical stimulation of the small intestine was applied during periods of sleep and wakefulness. Recordings and averaging of the evoked responses were performed with Spike 2 (CED) software package using the onsets of intestinal electrical stimulation as a trigger. A fragment of the EEG with the markers of electrical stimulation is shown in Figure 1. ERP obtained by averaging of the signal in 1 s time windows before and each stimulus across the entire recording session is presented in Figure 2.

Figure 2 demonstrates the first three waves of the averaged evoked response with maxima around 200 ms (P 200) and 500 ms (P 500), and a minimum at 340 ms (N 340) after stimulation. The range of ±2 standard errors of the mean (SEM) is shown as gray lines above and below the black line representing the mean value.

One can see that the first component P 200 and the second component N 340 clearly deflect from the 1 s background interval before the stimulation. However, for the third putative component P 500 the situation is not that clear and this component is closer to the ±2 SEM threshold of the background. For achieving the final conclusion regarding the presence of a particular ERP component in such “near threshold cases” we applied an additional criterion derived from permutation of the trial-by-trial mean values, as described below.

**FIGURE 3:**
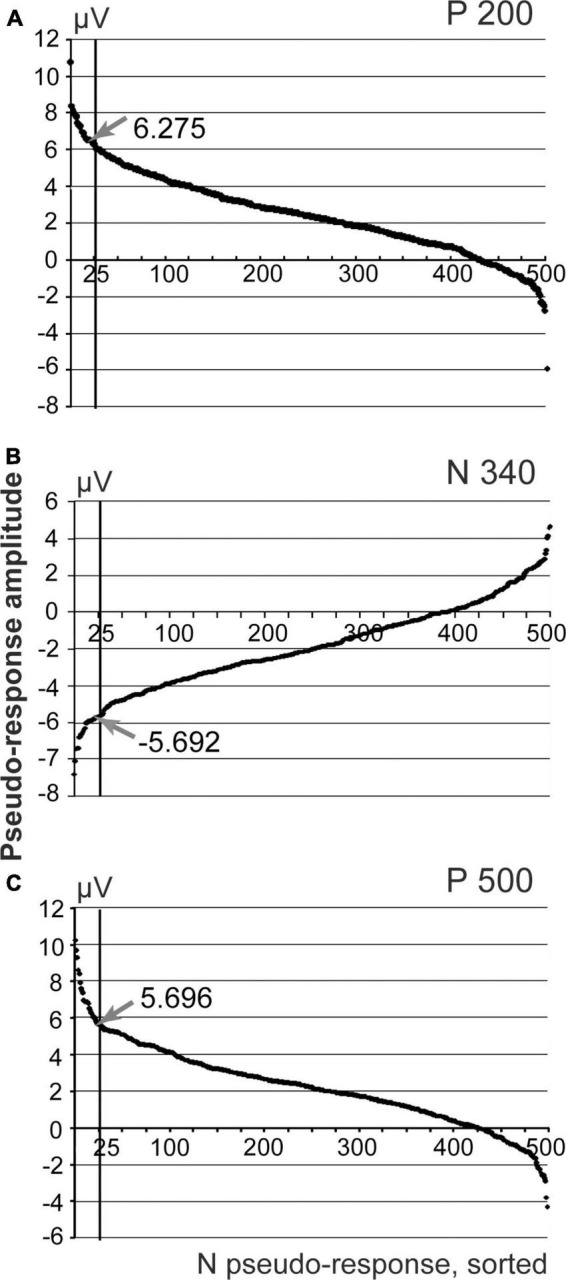
Sorted amplitudes of the averaged pseudo-responses, n permutations = 500. **(A)** Distribution of pseudo-responses for the interval corresponding to P200 component of the evoked response; **(B)** N 340 interval; **(C)** P500 interval. Arrows demonstrate the amplitude values obtained for the real evoked response.

Responses to 131 stimuli were averaged to obtain the evoked response shown in Figure 2. For the purpose of permutation-based analysis, the custom-made Spike 2 program generated the same number of markers randomly distributed over the same interval of the EEG recording. These random markers were used as triggers for averaging of the new pseudo-response. After that maximal deflection of this pseudo-response was measured within the time interval in question at the time point that corresponds to the maximal absolute value of the deflection (e.g., between dashed lines 3 and 4 for P 500 component in Figure 2). The same procedure of random marker generation and EEG averaging was performed 500 times and 500 maximal deflection values obtained. These values, sorted in descending order for positive components and in ascending order for the negative component, are presented as distribution curves in Figure 3. The confidence interval for detecting the presence of an ERP component was set at 95% of the values of such distribution. Thus, the value of the 25th point of the sorted distribution corresponded to *p*-value = 0.05. All components with amplitudes higher (for positive components) and lower (for negative ones) than the confidence interval threshold were considered significant. For example, for our P 500 component the threshold value was equal to 5.7 μV. The real P 500 amplitude was equal to 7.43 μV, which corresponded to *p*-value = 0.03. Therefore, P 500 component was considered significant. The other mentioned components were tested in a similar way and were significant as well.

For all significant components we performed further analysis aimed to investigate whether all of these components were evoked in response to every stimulus applied or only to some of the stimuli.

### Selection of the “efficient” trials

As mentioned above, procedure of the efficient trial selection was implemented as a script for Spike 2 version 6.16 to augment EEG analysis routines of this package, and was later re-implemented as a Matlab script (freely available).^1^

Estimation of the efficiency of the trials was done independently for each of the three significant components of the ERP obtained at the previous step. We further describe this procedure using the P 200 component as an example. The process was identical for the other components.

For this analysis we arbitrarily defined the time range where the component in question can be observed. For P 200 component that was the interval shown between dashed lines 1 and 2 in Figure 2. Usually the interval between two points where the ERP signal crossed zero line was chosen for further analysis. The shape of the ERP component located between dashed lines 1 and 2 was taken as the template for further comparison with the shapes of the individual waveforms for all 131 trials in the same interval from the trial onset. The algorithm evaluated Pearson correlation coefficient between each individual trial shape and the template resulted from averaging. The program considered only positive correlations; with negative values replaced by zero. Thus, every trial received a corresponding value of the correlation coefficient, either positive or 0. Sine similarity (S) was calculated as

*S* = 1–r^2^ for *r* > 0, and *S* = 1 for *r* < 0

were r is Pearson correlation coefficient. Pearson correlation value was squared to contrast the shape differences in order to make trial separation clearer, and 1− r^2^ was used to allow to intuitively interpret this formula as “difference”: the smaller the value is (ideally–something close to zero), the smaller is the difference between the average (the template) and the individual trial waveform.

In our early tests, the initial formula to compare the waveforms—the average and the trial—was


$$\frac{\int_{a}^{b}{\left(f\,\left(x\right)-g\,\left(x\right)\right)}^{2}\,dx}{b-a}$$

where f() and g() are waveforms compared on the interval (a, b)—or, in discrete form,


$$\frac{\sum_{i=1}^{N}{\left(f_{i}-g_{i}\right)}^{2}}{N}$$

where *i* = 1… N are the indexes in waveform arrays f and g. Here small values represent a good fit; however, we quickly realized that the amount of variation in the trial waveforms can be quite substantial, and therefore there is a need for a normalized criterion. Nevertheless, we preferred to keep the same intuitive rule—“smaller is better.”

As a result, sine similarity threshold varied from zero to one, but unlike the correlation coefficient, the lower value corresponded to higher likeness between the template and the individual trial activity, and the amount of selected efficient markers for lower thresholds was smaller.

Neuronal responses to stimuli are affected by the intrinsically probabilistic nature of the ion channel mechanisms. That normally results in a jitter in response timing. Thus, it might be beneficial to account for a jitter while computing Sine similarity. The implemented algorithm allows moving the template along the time scale within a defined small window until the best match is found between the individual response and the averaged one, to account for that jitter. The offered code provides such an option by introducing a *maxshift* parameter equal to a number of points of data sampling that can be chosen by a user to reflect the expected jitter. If maxshift > 0, S is calculated as many times as the number of maxshift data points surrounding the component marker, including 0 time that corresponds to the marker. The lowest trial value of S among the ones obtained using maxshift is chosen for the further described thresholding of the individual evoked responses.

**FIGURE 4:**
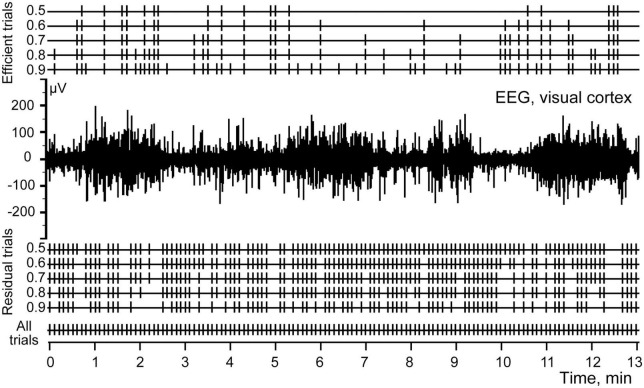
Efficient and residual trials for P200 component of the evoked potential, calculated at S thresholds ranging from 0.5 to 0.9.

Figure 4 shows five sets of markers selected for the P 200 component using different thresholds ranging from 0.5 to 0.9. Markers of efficient and residual trials are given above and below the EEG channel, respectively.

### Choosing the optimal threshold for trial separation

Since the above described procedure might seem arbitrary, it seems crucial to establish the criteria for choosing the best threshold. For that we compared averaged responses to the selected efficient markers with averaged responses to the residual markers. The main goal was to achieve the most prominent evoked response shape for the averaged efficient trials and flat averaged residual shape at the same time. The reasoning underlying that choice is the following. In the case when not all of the efficient trials are selected, after averaging of all residual trials we would still see some small response in the shape of the averaged residuals. To flatten the shape of the averaged residuals one needs to change the threshold in a way that produces more of the efficient and less of the residual trials. At some threshold the averaged residual signal should look as a uniform noise without visible peak at the expected position of the evoked response component. Changing the threshold further would lead to more of the noise to be included in the efficient trials. This would also start changing the sign of an averaged response for the residuals.

**FIGURE 5:**
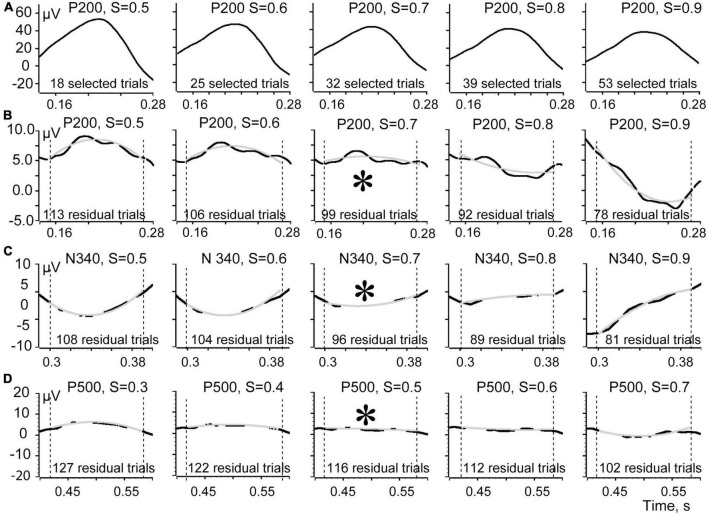
Shapes of the evoked response components averaged for efficient and residual trials. **(A)** Shapes of the P200 component averaged using efficient trials markers corresponding to S ranging from 0.5 to 0.9. **(B)** Shapes of the averaged residual signal for the corresponding thresholds for the P 200 component. **(C)** Averaged residual signals for N 340. **(D)** Averaged residual signals for P 500. Asterisks signify the S values corresponding to the least deflection of the averaged residual signal from 0.

This relationship of the efficient and the residual shapes is illustrated in Figure 5. In this figure we show short time intervals (140 ms) around the peaks of all three components of the evoked response. Shapes of the P200 component averaged using markers corresponding to different S threshold values ranging from 0.5 to 0.9 are shown at the top row (A). The shapes of this component look rather similar for all thresholds although slightly flatten from left to right. The averaged N 340 and P 500 components also demonstrated very little difference for different thresholds, and we do not present them in this figure. Row B shows the shapes of the averaged residual signal for different thresholds for the P 200 component. Rows C and D show averaged residual signals for components N 340 and P 500. For better visual estimation of the sign and the flatness of the averaged residuals these curves were “smoothed” using the “Fit data” tool of the Spike 2 program (gray superimposed curves). It is seen that for all our components the signs of the curvatures were reversing when S thresholds were increasing. Thus, the threshold of choice would be the first threshold that causes the curvature of the residual signal to reverse polarity or the one that leaves it flat. With that threshold the sum of all absolute values of the averaged residual component is minimal. Asterisks in Figure 5 highlight these choices. For the components P 200 and N 340 the best thresholds were *S* = 0.7. For the component P 500 the best threshold was 0.5. Final conclusion concerning the optimal threshold depends on the goal of a study–what is better, to take some “false” trials, or not to take some real ones.

**FIGURE 6:**
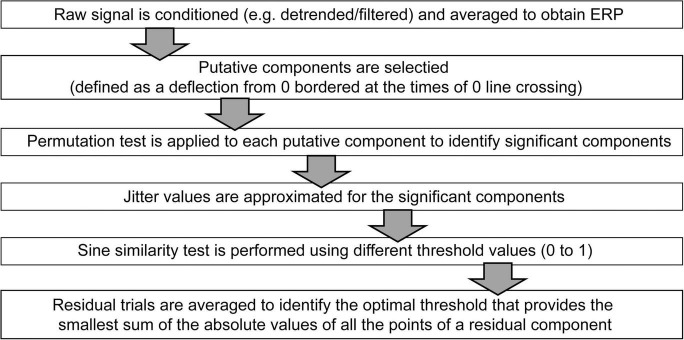
Workflow diagram of the method.

The above described procedure is summarized in a workflow diagram shown on Figure 6.

## Result

**FIGURE 7:**
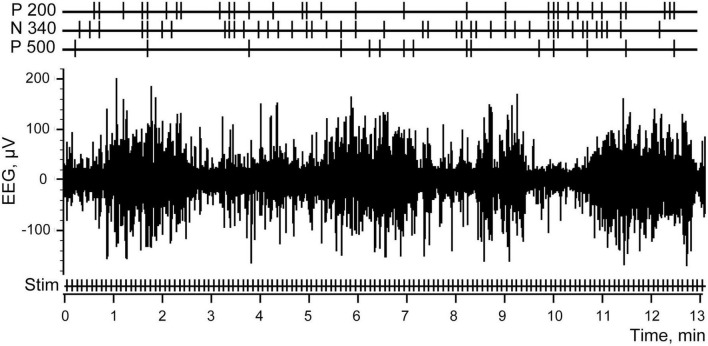
Electroencephalogram (EEG) and the stimulations of the abdominal viscera. Stim correspond to all electrical stimulations applied, P 200, N 340, and P 500 mark the efficient trials for each of the three components.

Figure 7 demonstrates the same fragment of EEG as in Figure 1, but with three channels added at the top, demonstrating the markers which indicated efficient trials selected for three components with the best thresholds as described above.

**FIGURE 8:**
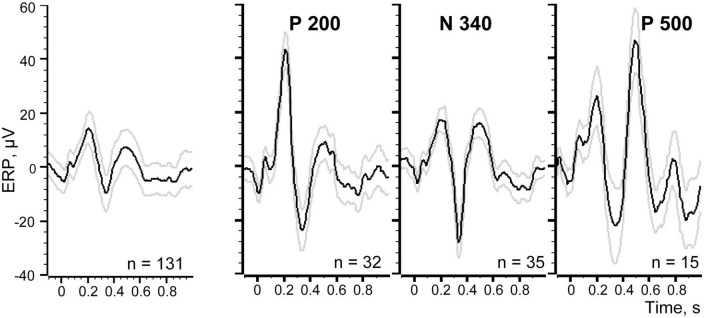
Evoked responses averaged using different sets of trials. Left panel demonstrates the averaged evoked potential for all 131 trials of the recording session.

In Figure 8 at the left panel we show the evoked response averaged for all 131 trials (identical to that shown in Figure 2). Three other panels of the Figure 8 demonstrate three evoked responses averaged using markers selected with the best S thresholds for each of the three components. All peaks of these components indeed became much larger, while the peaks of the other two components were reduced but still visible. This is not surprising because the positions of the markers selected for three components partly overlapped (Figure 7).

**FIGURE 9:**
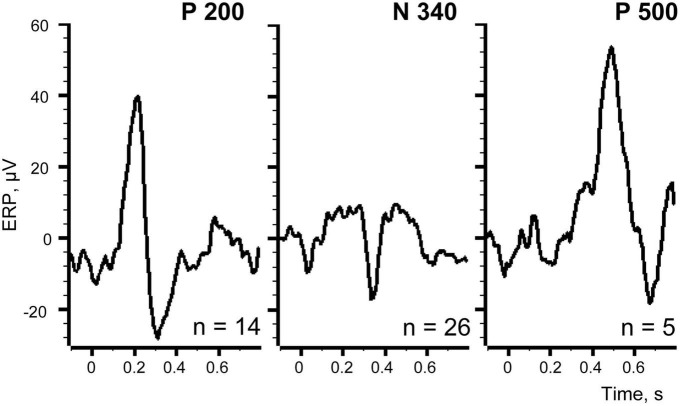
Averaged evoked responses for the trials where each of the components appeared alone.

We further selected markers for those trials where each one of the components appeared alone, excluding the overlapping trials. Using these markers as triggers for averaging it was possible to estimate “the pure shapes” of the investigated components. Figure 9 shows thus obtained pure shapes of our three components.

## Discussion

The method proposed in our article allows studying the presence of different evoked components in a signal coming from a single brain location at different time intervals. To make the most of this approach we always try to record activity of a well-localized brain area and commonly perform MRI imaging for our animals before starting a set of experiments to achieve a desired electrode placement. We also record intracranial EEG or local field potential (LFP) in a bipolar way from two microelectrodes with their tips located within several hundred microns from each other to ensure clarity of the origin of the recorded signal. The importance of local bipolar recording for some aspects of LFP analysis was described earlier (e.g., Herreras, 2016; Shirhatti et al., 2016). Thus, we can be fairly sure of the localization of a particular signal in the brain, however, the described variability of the responses still requires additional analytic approaches to further relate it to various internal or external influences that might underlie such variability. Although there are approaches developed for trial classification under similar conditions, they usually require training the classifier by presenting a set of stimuli having different parameters while local field potentials are recorded (e.g., Dezfouli and Daliri, 2020). However, such approaches are inapplicable to many situations when responses depend on the internally changing parameters, such the changes of the state of vigilance. In addition to that, training a classifier to certain stimuli might be difficult in experiments involving awake behaving animals, where recording session time is limited. Another method was suggested by Iannetti et al. (2005), and includes analysis of the amplitudes of the components of an averaged evoked potential, but the shape of the response or the response jitter were not considered.

Templating has been previously used in ERP analysis. Woody (1967) suggested the method of *adaptive filter*, an iterative approach which utilized maximum of covariation coefficient and might or might not converge to the correct waveform (Woody, 1967), when our paper proposes a method of components classification based on a threshold value of the Pearson’s correlation coefficient. The disadvantage of using covariation coefficients (correlogram) instead of Pearson coefficients is that the calculated values are not normalized and therefore do not generalize well.

The method of adaptive filter was primarily focused on finding in the signal responses with the known and well-defined shapes. That can be highlighted by the Woody’s choice of the test signals having stereotypical shape with low inherent variability such as ERPs recorded from anesthetized cats, spike-and-dome epileptic discharges and motor neuron spikes. Another methodological study relied on the same idea of “true” low variance evoked responses to reject artifacts (Talsma, 2008). However, growing interest to brain activity of awake behaving animals, the need to study cognitive function fluctuating in time, and to include state of vigilance into the picture necessitate developing methods which take into account the inherent high variability of ERP components in question. Our work addresses this issue. Rather than assuming the existence of some “true” ERP shape including all of its components we propose that neuronal assembles contributing to each component do not necessarily work together and might even compete, and that in some cases ERP might in fact be a result of averaging of different activities that need to be studied separately.

Our method can be applied not only to the analysis of EEG or LFP, but also to the analysis of neuronal spike trains. Neuronal spiking is often analyzed using average peristimulus histograms (the method has been in use since the 1970s, e.g., Henry et al., 1973), which are affected by response variability as well. For that analysis spiking activity has to be transformed to a spike density curve that can be used in an analogous way to the above described procedure. This can potentially be informative for detection of time intervals during which the studied brain area received information from a particular source of afferentation or was receptive to a particular stimulus.

In its current form the method requires supervision for making decisions regarding the shapes of the ERP components to investigate as well as to choose the threshold values. However, it can potentially be made unsupervised by setting up a criteria for a component as a significant deflection from zero with a particular polarity, choosing its boundaries as the points where the averaged ERP crosses zero line, and setting up the threshold as the one corresponding to the sum of residual trials with a minimal difference from zero.

The offered method is certainly qualitative and heuristic in nature. One cannot exclude that in some trials an accidental wave of noise resembling the real evoked response by shape could coincide with the stimulus, in which case the trial would be falsely selected as the efficient one. However, this problem always presents in any averaging procedure. On the other hand, proposed comparison of the averaged responses to selected and residual trials offers a reasonable way to estimate quality of the performed selection.

## Conclusion

We believe that the proposed approach can be useful for investigation of the causes underlying variability of the evoked responses since it enables selecting individual trials with the clearest contribution to the averaged ERP components for further analysis.

## Data availability statement

The original contributions presented in this study are included in the article/supplementary material, further inquiries can be directed to the corresponding author.

## Author contributions

IP, GF, EL, and MP were involved in discussing the project’s idea and obtaining funding. GF created the computer programs mentioned in the manuscript. IP and MP collected the data re-utilized for this project. AL and EL tested different variants of the programs during development. IP analyzed the data presented in the manuscript. All authors participated in manuscript preparation, editing, and final reviewing of it.

## Footnotes

1
https://github.com/george-fedorov/erp-correlations

## In memoriam

We dedicate this work to the memory of our tragically deceased colleague and friend Ivan N. Pigarev.
